# Factors influencing physical activity in adults with cystic fibrosis

**DOI:** 10.1186/s12890-021-01482-x

**Published:** 2021-04-02

**Authors:** Nicola Hurley, Niall M. Moyna, Bróna Kehoe, Noel McCaffrey, Karen Redmond, Sarah J. Hardcastle

**Affiliations:** 1 School of Health and Human Performance, Faculty of Science and Health, Dublin City University, Dublin 9, Ireland; 2 Department of Sport and Exercise Science, Waterford Institute of Technology, Waterford, Ireland; 3 ExWell, Chronic Illness Rehabilitation Centre, Dublin, Ireland; 4 Mater Misericordiae University Hospital, Dublin, Ireland; 5 Institute for Health Research, University of Notre Dame Australia, Fremantle, Australia

**Keywords:** Cystic fibrosis, Exercise, Physical activity, Psychology, Motivation, Psychosocial, Behaviour change

## Abstract

**Background:**

Physical activity (PA) is a well-documented and accepted adjunct therapy for the maintenance and improvement of long-term health in cystic fibrosis (CF). Although the benefits of PA for CF populations are well-established, adherence to PA programmes within this population remains low. This study aimed to investigate the factors that influence engagement in physical activity, and to explore exercise preferences, among adults with cystic fibrosis (CF).

**Methods:**

Semi-structured telephone interviews were conducted. Participants were twenty-one adults (mean age 35 years, SD ± 8) with an established diagnosis of CF, living in Ireland. Interview scripts were digitally recorded and transcribed verbatim. Thematic analysis was used to analyse the data.

**Results:**

Four main themes emerged: *barriers, motives, value of exercise-related outcomes, and exercise preferences*. The main barriers included: low energy levels, time, the weather, and exercise-related confidence. Enjoyment and perceived competence underpinned autonomous motivation. Participants who self-identified as being regularly active valued personally identified exercise-related outcomes such as, accomplishment and affect regulation. Participants indicated a preference for home-based physical activity programs compared to gym- or facility-based programs.

**Conclusion:**

Interventions aimed at promoting physical activity among adults with CF should involve programs that foster autonomous motivation, enjoyable activities, personally identified outcomes, competence and that can be conducted from the home environment.

**Clinical implications:**

To increase physical activity participation among adults with CF, interventions that can be conducted from the home environment, that pay attention to the patients’ personally-valued exercise outcomes may be required.

**Supplementary information:**

The online version contains supplementary material available at 10.1186/s12890-021-01482-x.

## Introduction

Cystic fibrosis (CF) is a progressive, multi-system, inherited condition affecting approximately 1400 people in Ireland, and more than 70,000 worldwide [1, 2]. Today, with advancements in early diagnosis and the development of highly effective CF transmembrane regulator modulator therapies, people with CF are living longer [3, 4]. As such, increased emphasis is being placed on improving lifestyle-related behaviours in order to enhance long-term health in adults with CF [5].

Aerobic fitness has been shown to be an important predictor of survival in individuals with CF [6]. Those who have a higher aerobic capacity (VO_2_ ≥ 82% predicted VO_2_max) have an increased survival rate of 83% at 8-years, compared to 51% and 28% survival with moderate (59–81% predicted VO_2_ max) and low fitness (≤ 58%), respectively [7]. In a recent systematic review and meta-analysis including six studies (2019), Vendrusculo et al. [8] have shown that lower VO_2_max levels are associated with an increase of 4.9 in the risk of mortality in People with Cystic Fibrosis (PWCF). Evidence suggests that there is a positive relation between increased physical activity (PA) at moderate intensity and improvements in aerobic capacity, independent of improvements in muscle strength and pulmonary function [9, 10]. Regular participation in physical activity (PA); performing 150-min/wk, and preferably 300-min/wk of moderate-to-vigorous PA [1] improves a myriad of outcomes in CF, such as exercise tolerance [11], airway clearance [12] energy levels [13] and quality of life [14]. Sustained PA also has the potential to slow the annual rate of decline in pulmonary function [15]. Despite the established benefits of PA for individuals with CF, adherence to PA programs within this population can be poor [15, 16]. In order to develop the optimal PA program for individuals with CF, a better understanding of patient attitudes towards PA is needed to elicit sustainable lifestyle behaviours [5].

Few studies have investigated the factors influencing exercise participation in adults with CF [5, 16–19]. The most commonly reported motivators for sustained PA participation included enjoyment [17], motivation [17], improving general and/or lung-health [16], and feeling healthy [16]. Feelings of breathlessness [17], fatigue [18], lack of good health [5], reduced energy [5], and embarrassment when exercising in public [18] were among the most common barriers reported. Myers et al. [19] suggested severity of CF lung disease and being female were associated with more significant and rapid decline in lung function, as factors that may contribute to poorer long-term adherence to PA. Lack of motivation and time were also reported as barriers to exercise [5, 17].

Several models have been developed to provide a more thorough understanding of the relation between PA and behaviour change. The theory of planned behaviour focuses on beliefs and attitudes and social norms [20], Bandura’s social cognitive theory [21] focuses primarily on self-efficacy and outcome expectancies, and the self-determination theory [22] focuses on the quality of motivation and the importance of satisfying key psychological needs such as autonomy and competence-relatedness. To date, there is no optimal model to explain PA and behaviour change, and qualitative research has therefore been helpful, by not taking a deductive approach and approaching the topic with a broad perspective to unravel which dimensions appear to be most important [23]. The current study is not aligned to any particular theoretical framework as it is inductive in nature, and will explore the dimensions that influence PA behaviour change in a CF population, and therefore provide an insight into which behaviour change models may be most suitable when designing interventions. Given previous research findings and the aforementioned theories, it is expected that low self-competence and motivation may be theoretical components of emerging barriers to exercise in the CF cohort [23].

The purpose of this study was to explore the attitudes towards, and dimensions influencing, PA participation in Irish adults with CF. Such knowledge concerning the dimensions influencing PA and attitudes towards PA would be beneficial in the development of appropriate intervention strategies to promote PA in practice.

## Materials and methods

The current study conformed to suggested guidelines by Clark in appropriateness of method, transparency of procedures, and soundness of approach [24].

### Participant recruitment

Convenience sampling was used to recruit adults with an established diagnosis of CF (confirmatory diagnostic sweat chloride test). An email including a patient information leaflet was circulated by CF Ireland, the national CF charity, to all adults with CF on the registry in Ireland (n = 861). Following an expression of interest, participants were screened for eligibility. Participants were eligible for inclusion in the study if they were ≥ 18 years of age and living in Ireland. Written informed consent was obtained in accordance with the Dublin City University (DCU) Research Ethics Committee (DCUREC/2018/141).

### Data collection

Semi-structured telephone interviews lasting up to 30-min were conducted. An interview guide was developed based on previously published work (see Additional file 1) [25], regarding the factors that may influence PA in adults with CF. Interviews were digitally recorded and transcribed verbatim. Interviews were conducted in the Autumn of 2019 (September–October).

### Data analysis

Data were analyzed inductively using thematic analysis, which involved several steps [26]. The first step involved active and repeated reading of the transcripts to gain an insight into the data, and to explore initial codes. The second step involved systematically (i.e., line by line) coding features of interest within the data (data extracts) that are related to the research question. The third step involved exploring whether codes may be collated to form over-arching themes. This step, which re-focuses the analysis at the broader level of themes, rather than codes, involves sorting the different codes into potential themes, and collating all the relevant coded data extracts within the identified themes. A theme captures something important about the data in relation to the research question and represents some degree of patterned response within the data set. Confidence of the existence of a theme is established when a number of instances of the theme are identified across the data set or across the sample [26]. The fourth step involved reviewing and refining the potential themes. This phase involved checking whether there was sufficient data to support the existence of a theme or whether there was overlap such that themes might collapse into each other (e.g., two apparently separate themes might form one theme). In step four, the researchers’ checked for coherence between the data and the themes and checked for identifiable distinctions between themes. The final step involved the refining and defining of themes. Once a satisfactory thematic map of the data was achieved, themes were further refined and defined to be presented for analysis and cross-checked the data extracts within them. During this step, theme and sub-themes were reviewed to ensure that they matched the data and that each theme is coherent and internally consistent (i.e., not too much overlap between themes). As part of the refinement, decisions were also made concerning hierarchy and the presence of sub-themes that fit into a broader overarching theme. Sub-themes are used for giving structure to a particularly large and complex theme, and also for demonstrating the hierarchy of meaning within the data.

A second researcher experienced in qualitative data analysis served as a second reader of the transcripts and offered further insight with respect to emergent themes to broaden data interpretation and assist with coding, reviewing and refining of themes. Data saturation was determined following analysis and occurred at the point when no new information was gained to develop or broaden themes [27]. Credibility and rigor are demonstrated in the analysis in the following ways; (1) using data saturation to determine sample size, (2) describing in detail the steps taken in conducting the analysis, (3) involving two researchers in the process of analysis thereby broadening the number of interpretations and (4) providing ‘thick description’ via the use of extensive and direct quotations so that the reader can evaluate the interpretation (i.e., the fit between data extracts and the theme labels) [28].

During the thematic analysis process, the researcher ensured that each transcript was labelled according to self-reported activity status, using ‘low-active’ defined as not meeting the current consensus CF PA guidelines, ‘moderately active’ defined as meeting, or likely to be meeting, the guidelines, or ‘highly active’ defined as exceeding the minimum PA guidelines for PWCF, on the front page to identify potential differences between responses according to activity status.

## Results

Twenty-one adults (57% males) with an established diagnosis of CF participated in the study. The mean ± SD age of subjects was 35 ± 8 years, with 16% of participants having undergone lung transplantation. Participants’ pseudonyms, characteristics and demographics are outlined in Tables 1 and 2. Table 3 displays participant characteristics compared to the Irish CF population, and indicates that the sample in the present study is broadly representative of the Irish CF population in relation to gender, BMI and presence of at least one copy of the Δf508 gene. However, participants in the present study were older (median 34 years vs 21 years), with higher lung function (89% vs 82.3%), and a greater proportion had received a lung transplant (14% vs 5%).

**Table 1:** Participant pseudonyms and individual characteristics

ID code	Pseudonym	Gender	Age	Self-reported PA	transplant status
1	Paul	Male	30	High	No
2	Mary	Female	30	Moderate	No
3	Jenny	Female	44	Low	Yes
4	William	Female	34	Low	Yes
5	Richard	Male	49	Low	No
6	Matthew	Male	28	High	No
7	Shane	Male	33	High	No
8	Robert	Male	41	High	No
9	Emily	Female	50	High	No
10	Dorothy	Female	29	Moderate	No
11	Carol	Female	36	High	No
12	Kevin	Male	33	High	No
13	Amy	Female	32	Low	Yes
14	Eric	Male	50	Low	No
15	Brenda	Female	35	Moderate	No
16	Frank	Male	26	High	No
17	Peter	Male	19	Low	No
18	Keith	Male	38	High	No
19	Hannah	Female	36	Low	No
20	John	Male	31	High	No
21	Jason	Male	35	Low	No

**Table 2:** Participant demographics

FEV_1_ (% predicted)	84.1 ± 19.7
BMI (kg·m^2^)	24.1 ± 2.4
Diabetes Status	CFRD (n = 2)
Pancreatic Status	Pancreatic Insufficiency (n = 5)
Co-existing Conditions	Neurocardiogenic syncope; Osteopenia; Asthma; Nasal Polyps; CF Liver Disease; Gastro-intestinal Reflux; CF-related Arthritis
Homozygous ΔF508	4
Heterozygous ΔF508	3
Other CF Alleles	G542x; R117H; E60x
Location of CF Centre	Dublin (n = 15) Cork (n = 3) Limerick (n = 2) Galway (n = 1)

Results are reported as mean ± SD

Results for FEV_1_, BMI, CF Alleles, Diabetes and Pancreatic Status for 9/21 participant

**Table 3:** Study participant characteristics compared to the Irish CF population

	Study CF population	Irish CF population
Age (Years)	Median: 34	Median: 21
Gender (Males: Females)	42%: 58%	42%: 58%
BMI (kg·m^2^)	Median: 23.4	Median: 22.3
FEV_1_ (% predicted)	89	82.3
Genotype (at least 1 copy of Δf508)	87.5%	91.7%
Transplant Status	14%	5%

Data for BMI, FEV_1_ and Genotype are only available for 8/21 participants. Data regarding the Irish CF population was obtained from the CF Registry in Ireland Annual Report for 2019

**Fig. 1:**
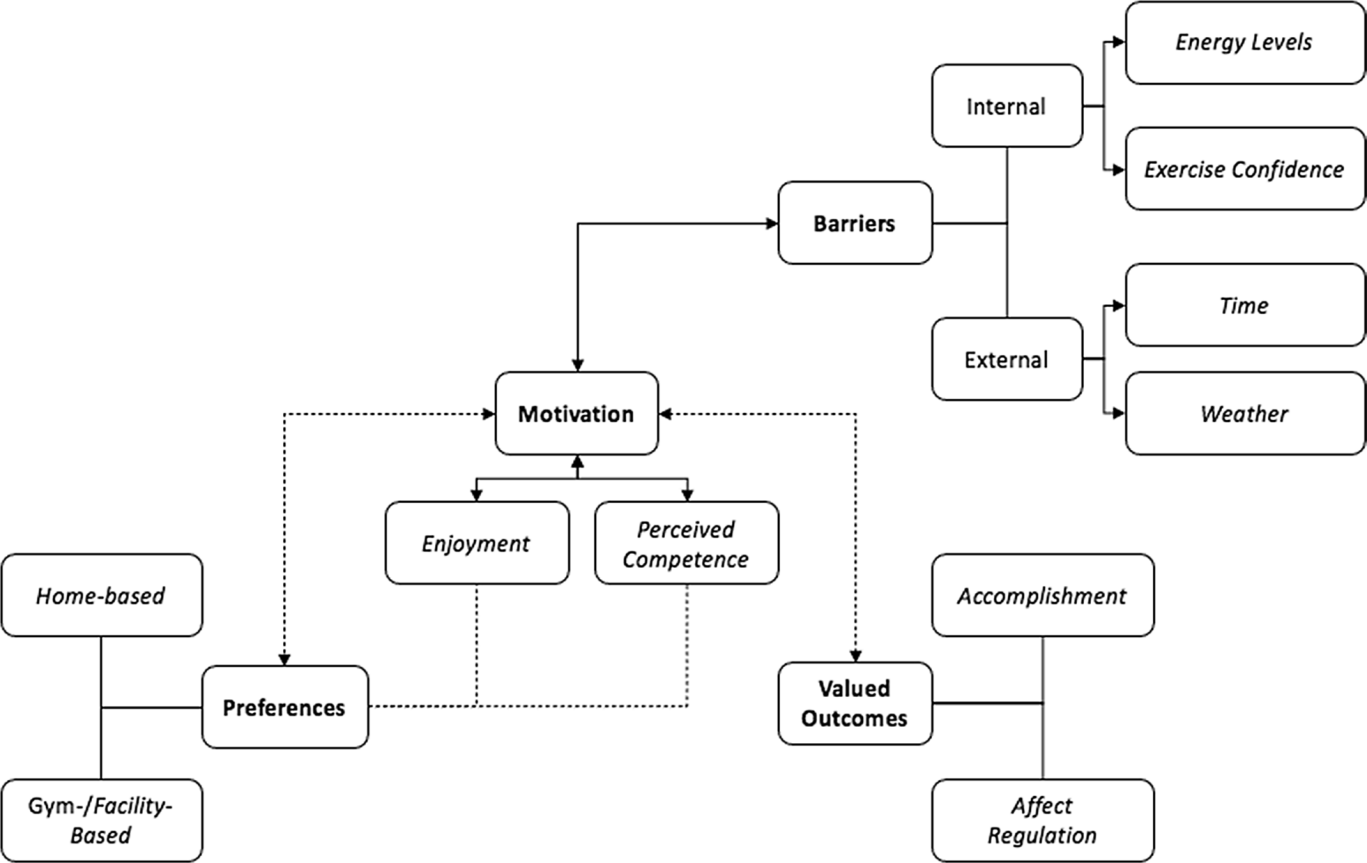
Thematic Map Displaying Themes and Relationship between themes. The thematic map displays the four primary themes (motivation, barriers, valued outcomes and preferences), and sub-themes. The arrows provided reflect the relationship between and within themes. For example, an individual who values exercise outcomes (through feelings of accomplishment or through affect regulation) is more motivated to exercise. Enjoyment of exercise and perceptions of competence affect motivation for PA and also preferences

Data analysis identified four key themes relating to factors that influence PA engagement in adults with CF: (1) barriers underpinned by four subthemes including energy levels, time, weather and exercise-related confidence; (2) motivation with sub-themes of enjoyment and perceived competence; (3) valued outcomes, underpinned by sub-themes of accomplishment and affect regulation; and (4) preferences (refer to thematic map in Fig. 1). Each quote is followed by a pseudonym and the individual’s age. To further contextualize the data, self-reported PA and additional quotes are provided in Tables 3 and 4. In accordance with Sandelowski (2000), pronouns will represent indeterminate quantities where ‘many’ implies approximately 75%, ‘several’ implies approximately 50%, and ‘few’ implies approximately 20% of the sample [29].

**Table 4:** Overview of themes and illustrative quotes

Theme	Illustrative quotes
Low energy levels were dominant internal barriers to PA engagement	“It’s just because I know it will be tiring” (Jenny, 44)
	“Just when I’m feeling unwell, lack of energy. Most days I don’t be in the mood to go to the gym, just through fatigue and tiredness” (Robert, 41)
	“Low energy, if I’m not feeling the best, I’d use that as an excuse” (Emily, 50)
	“Some days I’m just absolutely wrecked, like I can be so tired. I just don’t want to move, just don’t want to go out, sometimes I don’t even want to sweep the floor in the house, it’s that type of…just comes out of nowhere” (Paul,30)
	“Energy wise, yeah, I find my energy levels are even when I wake up in the morning are low” (Keith, 38)
	“I have just started college and on the days where I'm in, I know that I don’t have the energy to work out and a full day in college on the same morning” (Dorothy, 29)
Time and the weather were dominant external barriers to PA engagement	“it’s probably like time and weather” (Frank, 26)
	“I suppose time is one thing, obviously having CF and working full time and family it’s tough” (Keith, 38)
	“Time is definitely one as well” (Hannah, 36)
	“I work kind of in the evenings times 3 to 11 at night. So going to the gym I have to be up early to kind of go in and get to the gym early. And I have no problem with doing that, it’s just at times you wake up and you feel so exhausted from work that it’s just too difficult. Whereas if I had a schedule around 9 to 5, I’d go to the gym on the way home I’d do it more often” (Jason, 35)
	“The weather is getting bad, so, going to be stuck in doors more, will be hard to get up there, the motivation might slack a bit” (Paul, 30)
	“My motivation if the weather is [bad], my motivation is squat” (Jenny, 44)
	“As I say that’s weather depending” (Richard, 49)
	“The Irish weather I find is a barrier for me to get to be physically active” (Matthew, 28)
	“I don’t walk in torrential rain and… the winter months” (Jenny, 44)
Positive past PA experiences were typically associated with more regular PA participation, driven predominantly by enjoyment	“Positive, from the age of about, when I was in school and stuff, and I was always playing sport, I was always playing football” (Robert, 41)
	“Positive, I always try to keep active…I have always kind of done it throughout my life” (Emily, 50)
	“So, as a kid, I wouldn’t have noticed any difference from anyone else, I don’t think. Very active” (Hannah 36)
	“So, if I go for a run first thing in the morning it helps me clear all the mucus and stuff in my lungs. Then the afternoon I go to the gym for an hour weight session. And I do that for 2 days and then I have the third day off. And then I go through all that again.” (Robert, 41)
	“I usually try and go to the gym 3 mornings a week and that would include 2 spinning classes and a hit class, high intensity class. So they would each last approximately 30 min each but they would be quite physical.” (Emily, 50)
	“Yes, so at the minute I am exercising probably 5 or 6 days a week…I would run on the treadmill for about 10 or 15 min depending on how I'm feeling between 6 to 7.5 miles, that’s like speed wise. So, then I’d do some weights I do the sit ups” (Dorothy, 29)
	“I've always really enjoyed the exercise.” (William, 34)
	“I do enjoy the physical aspect of it, I enjoy putting my music on and just running the treadmill” (Dorothy, 29)
	“it’s fun and it’s enjoyable” (Carol, 36)
Value of exercise-related outcomes: accomplishment and affect regulation	“I enjoy kind of the buzz you get…the way it clears your head…you kind of feel better afterwards” (William, 34)
	“I like the buzz you get and the feeling after that you have accomplished being able to physically do an activity, everyone in the room…they don’t know I have mild CF and I kind of look and say, god I am able to keep up with them” (Robert, 41)
	“Sometimes it is the best medicine for you, you go off burn some calories, get a sweat on and you do feel the better for it” (Emily, 50)
	“I like the benefits of it, the immediate effects of it and the long term effects, just keeping the lungs healthy I have to say that’s my main benefit from it” (John, 31)
	“The great feeling you have after doing some activity really that you feel you got loads of energy, you feel like you have actually done something healthy” (Jason, 35)
	“Makes you feel good, even if you are stressed out at home or you had a bad day… just relieving that stress you have, even if you are tired and you think ah can’t be bothered… you always feel better after it and you say you are kind of glad [you] went” (Robert, 41);

### Barriers

Participants reported both internal and external barriers to PA participation. The most common internal barrier was low energy: “There are some days where I’m just really mentally drained…So, the idea then of having to get up on a bike can be a huge barrier because I just feel so exhausted” (Amy, 32); “I’ll just be generally really tired…so that might get in the way of being motivated, I don’t want to push myself (Dorothy, 29); “Energy would be a big one. Sometimes I’ve just enough energy to get up and do my nebs, and I don’t really have the energy to do other things” (Hannah, 50). Participants also reported barriers pertaining to exercise-related confidence, such as self-consciousness: “In gyms and public when you’re coughing, there’s people looking at you to say what is wrong with you?” (Eric, 50), and concerns of capability; “The thing that turns me off PA is the fear of not being able to do [it], if that makes sense? Everyone in the class is jumping up and down on the step and I’m like oh my God, I can’t keep up” (Hannah, 36).

External barriers to regular participation in PA included time: “I just generally don’t think I get the time” (Mary, 30); “Essentially it’s time. Time is the major one for me… prior to the kids…I was getting much more exercise, but time is the main thing” (Richard, 49); and the weather: “The weather here in Ireland is just…you could plan to go and have a round of golf, or play tennis at the weekend, and then it’s raining all day so that stops that” (Matthew, 28); “If the weather is bad, my motivation is squat. I’m very much a fair-weather walker, you know?” (Jenny, 44).

### Motivation

Motivation was a dominant theme identified throughout, with several participants displaying higher levels of motivation: “I’m always very self-motivated actually, I suppose from a young age I always kind of realised that…, it’s me that’s going to do it, nobody else can do it for me” (Amy, 32); “I’m very self-motivated, I don’t need somebody shouting at me in a gym” (John, 31), compared to others who were primarily motivated by external drivers, such as guilt: “It’s more guilt than anything I have to say. I go through stages of doing it but it’s guilt more than anything else” (Eric, 50); and “It’s hard to keep motivated because you know you have to do it” (Mary, 30). Enjoyment and perceptions of competence appeared to underpin PA motivation.

### Enjoyment

Participants who expressed positive attitudes towards and enjoyment of PA: “It’s always been a positive, I have been really active most of my life now” (Kevin, 33); “Yeah, very positive. I feel the more exercise I do, the better I feel” (Richard, 49), seemed to engage in more regular PA: “I go to the gym five days a week; I do twenty-five minutes of cardio and then I do one particular muscle”” (Kevin, 33), and “I’m very active, I’d get up at 7 o’clock I go for a 3 km run…then in the afternoon I go to the gym for an hour weight session. And I do that for 2 days and then I have the third day off…then I go through all that again” (Robert, 41). Exercise enjoyment appeared to be a motivating factor for adherence: “I think the main thing is to do something you enjoy. Anytime I’ve been successful at kind of maintaining sport, it’s just doing something I enjoy” (William, 34); “I suppose …the main reason is, I enjoy it” (Matthew, 28).

In contrast, some participants reported a lack of enjoyment as a reason for their physical inactivity: “None of it. I’m going to be very honest with you, I do not enjoy PA at all” (Eric, 50); “To be honest, I don’t particularly enjoy it!” (Mary, 30); “Mainly, I just don’t like exercise” (Peter, 19), which was typically underpinned by poor engagement: “At the moment I would say my PA is pretty much zero… I haven’t been doing much exercise” (Peter, 19), and/or negative past experiences with PA: “Very negative. For me, PA was just hard” (Jenny, 44); “I suppose negative. I wouldn’t be going to the gym. I wouldn’t be interested in any of that” (Eric, 50).

### Perceived competence

Several participants reported high levels of perceived competence that appeared to underpin their exercise motivation: “You know, even though I have this illness, I never really felt like I did because it really didn’t keep me from setting records on the leader board when I was younger…I was very fast” (Carol, 36): “But it’s just the realization that I am as fit as most of them in the room and it’s at that… and keeping that level up, it’s keeping me on par with most of them in the room and for my own benefit as well” (Robert, 41); “I know I’m well able to do it” (Emily, 50).

Other participants did not identify as being the sporty or exercise type: “I’ve probably never been that person. I was never a sporty person” (Jenny, 44); “Exercise wouldn’t be in my remit. I wouldn’t be interested in any of that. I wouldn’t be a gym bunny; I’d be far from it. As far away from it as you can imagine” (Eric, 50), and expressed low perceived competence in their ability to perform PA: “I’ve been thinking a lot about this, and it’s not being able to do it. The thing that turns me off PA is the fear of not being able to do the activity if that makes sense” (Hannah, 36).

### Valued outcomes

Many participants who perceived that they were regularly physically active reported exercise-related outcomes, such as accomplishment and affect regulation as drivers for continued engagement in PA. Many of the participants identified with the importance of keeping healthy: “I mean, just being healthy and not getting sick. Feeling good…and my lung functions being good, that motivates me” (Brenda, 35); “To try and stay as healthy as possible is a motivator” (Richard, 49); “I just realise that if I want to be healthy and if I want to live a kind of normal life, I have to do this…so that pushes me to keep myself as healthy as I can be” (Robert, 41).

Several participants were reportedly driven by a sense of accomplishment: “I do like it in the end…when you feel like you have accomplished something” (Mary, 30); “I like…the feeling after that you have accomplished being able to physically do an activity” (Emily, 50). Affect regulation emerged as a key influencing factor among participants who regularly engaged in PA: “I like how it makes me feel good and I’m always in a better mood, like it releases endorphins and I’m a happier person afterwards definitely” (Brenda, 35); “Just how you feel after doing them, you just feel well and it’s good for everything, not just CF, but your mental health and everything…whether you are sick or not, you do feel great about yourself” (Paul, 30).

### Preferences

Many participants reported a preference for exercising within their home environment, as opposed to the gym: “I love doing my treadmill and stuff at home…it reduces the risk of infection as well” (Mary, 30); “At home, just because it’s less time consuming and less daunting… it’s easier than going to the gym…if your treadmill is sitting there looking at you” (Dorothy, 29); “Home-based…like it can be stressful for me to go to the gym…it’s germy and noisy and if you don’t feel well you kind of just want to leave once you get there” (Carol, 36).

The concept of attending a gym was perceived negatively by a large number of participants, due to the risk of cross-infection: “They say the worst place to pick up germs that are bad bacteria is in the gym, because everyone is in there sweating. And people that feel sick still go to the gym and they are coughing all over the place and sweating and that kind of puts me off” (Robert, 41); “Going to anything like a gym I just have total war against…I think they’re a hive of infection. I think they’re the most unclean environments you could possibly get”, and illness-related embarrassment: “I don’t like when people listen to me coughing when I’m in the gym…and if I have any secretions, at least if I’m at home, I’ll get rid of it there” (Mary, 30); “Going to the gym, especially on Oxygen and things is quite intimidating for people…it’s very easy to feel self-conscious so, I think home programmes for people… might help” (William, 34).

Many participants highlighted the Exercise Grant, made available biannually by CF Ireland, as a beneficial tool for enabling home-based exercise: “I have a treadmill at home that I was able to get through the CF Ireland Exercise Grant, which is fantastic, and that is what I would use at home. And, I have got a few little weights…it’s easier for me than driving to the gym, which takes more time out of my day” (Emily, 50), and “From a CF Ireland point of view, the Exercise Grant is really good because it can kind of help with the financial burden of exercise” (William, 34).

## Discussion and conclusion

### Discussion

The present study is one of the first to explore factors influencing PA in adults with CF. The main barriers among participants were internal (*low energy*), external (*time* and *the weather*) and related to exercise confidence (*self-consciousness* and *capability*). The findings of the current study are consistent with previous research which found that the lack of energy and time, and bad weather contributed to poor PA compliance among adults with CF [5, 17] and COPD [30]. Exercise-related confidence as a barrier to sustained PA engagement among adults with CF is a novel finding.

Motivation was a dominant theme underpinned by enjoyment and perceived competence, and appeared to differentiate between those that self-reported being more and less physically active. Specifically, those who reported positive past experiences with PA, higher levels of perceived competence, and more autonomous forms of motivation appeared to engage in more frequent and sustained PA. In contrast, those who reported negative past experiences with PA and lower perceived competence appeared to be less physically active, linked to feelings of external or introjected regulation. Our findings are consistent with the self-determination theory (SDT) in relation to the innate psychological needs (specifically competence and autonomy) that must be satisfied in order to be autonomously and more intrinsically motivated [22]. According to SDT, controlled motivation is when individuals are motivated in order to satisfy some form of external pressure (e.g., physician) or internal pressure (e.g., sense of guilt). Some participants in the current study that were notably less motivated to exercise reported motives driven by extrinsic factors and reported participating in PA to avoid feelings of guilt (introjected regulation). More autonomous motivation is achieved when the individual values health or physical fitness, or when the behaviour (PA) is consistent with his or her ambitions in life or identity (identifying with valued outcomes). Indeed, the present study found that valued exercise-related outcomes were drivers for continued exercise participation amongst participants who appeared to be more regularly physically active. Consistent with SDT and previous research, more autonomous motivation rather than controlled or externally driven motivation are associated with regular exercise behaviour [20, 21]. The strongest predictor of exercise maintenance is personally valued outcomes [31]. Participants in the present study who self-reported to be more regularly active reported affect regulation as a motive for continued engagement in PA. The role of exercise for affect regulation is a novel finding.

A key finding was that most participants, particularly those who were more motivated, expressed a preference for home-based, rather than gym- or facility-based PA. This is notable, since home-based interventions have the capacity to potentially ameliorate feelings of low perceived competence, self-consciousness and remove barriers associated with time and changing weather [22]. It is possible, also that the wider CF population, likely to be less interested in exercise, are unlikely to prefer the gym. It should be acknowledged that the present sample was of an older age profile than the wider Irish CF population, and this may have influenced exercise preferences. Younger CF patients may prefer facility-based exercise, and further research is required to further elucidate exercise preferences in the broader CF population. Tailoring PA programmes according to patient preferences and psychographic profiling [28] may optimise uptake and adherence.

### Practice implications

The reported barriers to PA may be modifiable. For example, self-consciousness and low perceived competence could be mitigated through the provision of mastery experiences to provide a sense of accomplishment and competence. Clinicians could be trained in assisting patients with effective goal-setting, action planning and problem solving for physical activity to provide a sense of accomplishment and alleviate barriers. Home-based interventions that focus on PA rather than facility-based exercise or sport may help alleviate feelings of self-consciousness and low energy. Clinicians could also be trained to help foster autonomous motivation by asking patients to describe some personally valuable outcomes of exercise participation, in addition to providing a rationale for the health benefits of exercise to CF patients.

Several systematic and meta-analyses have highlighted motivational interviewing (MI) as an approach that has been shown to be effective in changing health behaviours [32]. Providing healthcare professionals with additional training in the use of activity counseling, brief motivational interviewing and techniques to foster the increased importance of PA and confidence to exercise (for example, the use of importance and confidence rulers in brief MI), may have the potential to increase autonomous motivation, and decrease external regulation, toward long-term engagement in PA among patients with CF [33] and has been recommended in other patient cohorts. Motivational interviewing is advantageous in the clinical setting because it is an individualized intervention appropriate to all patients along the continuum of motivation. For those CF patients that are less motivated to change, strategies can focus on raising the importance of physical activity and exploring confidence to change. For those patients more ‘ready’ to change, more action-oriented strategies such as goal-setting and action planning can be implemented [31]. Individual barriers and exercise preferences can also be explored to devise a patient-centered intervention.

Given the findings in relation to participants’ motives and exercise preferences, home-based interventions may hold promise in addition to overcoming the barriers associated with facility-based exercise programmes, such as the fear of exercising in public and the risk of cross-infection. Interventions aimed at developing feelings of competence and a sense of accomplishment (via goal setting) may facilitate increased adherence as they have been found to underpin autonomous motivation. Prior to the development of exercise interventions in this group, further research is needed to confirm or expand the exploratory findings of the present study. Future research that ascertains exercise perspectives of those CF patients less interested in physical activity would be worthwhile. In addition, it would be worthwhile to canvass attitudes towards physical activity engagement amongst CF patients experiencing symptom exacerbations and/or more severe disease progression.

### Study limitations

The study was limited by the relatively low uptake which may not be representative of the wider CF population in Ireland. The absence of exclusion criteria and discussion surrounding the presence of symptoms must also be noted as a limiting factor and the study did not explore whether other comorbidities influenced the results. A further limitation is the absence of objectively-derived PA data for the sample. Opportunistic sampling may have resulted in the recruitment of a more physically active that recognise the benefits of regular PA participation, overlooking the perceptions of those who are disinterested in PA. Although the sample appeared to be similar to the Irish CF population in relation to gender and BMI, lung function was higher in the present study which may indicate that we have recruited participants that are healthier and may have more positive attitudes towards PA. The sample was also older, with a higher proportion having had a transplant which could partially account for the preferences for home-based PA. The findings in the current study are tentative, and it is unclear as to whether they are transferable to the wider CF population. Further research is required to comprehensively evaluate the factors influencing PA among adults with CF. It is also important to acknowledge the relevance of our findings in relation to the Covid-19, and post-Covid-19, environment, highlighting the value of home-based exercise for vulnerable populations, such as those with CF.

### Conclusion

Barriers to PA engagement in the current study were low energy levels, time, the weather, and exercise-related confidence. A tentative but important finding was the preference for home-based rather than gym- or facility-based PA. Effective interventions will likely require the promotion of autonomous motivation, enjoyable activities, personally identified outcomes and competence among adults with CF.

## Supplementary information

**Additional file 1**. An interview guide was developed based on previously published work.

## Data Availability

Data will be made available upon reasonable request.

## Abbreviations

CF: cystic fibrosis
PA: physical activity
