# Supplementary material for: Factors influencing physical activity in adults with cystic fibrosis
Source: BMC Pulm Med. 2021 Apr 2;21:113. doi: 10.1186/s12890-021-01482-x (PMC8017094; doi:10.1186/s12890-021-01482-x)
Supplement: Supplementary file 1 — Additional file 1. An interview guide was developed based on previously published work. [file 12890_2021_1482_MOESM1_ESM.docx]

| **INTRODUCTION** | |
| --- | --- |
| Hi, my name is Nicola  Thank you very much for agreeing to participate in this telephone interview.  Today, we are going to talk about the topic of physical activity with respect to cystic fibrosis – more specifically we will touch on aspects such as your current physical activity levels, what motivates you to participate in physical activity, the challenges that you face that may prevent you from participating in physical activity and finally, we will discuss strategies that you think may improve enjoyment levels, in turn, enhancing participation in physical activity and physical activity programs.  So, we would like you to be as honest as possible during this interview, there is absolutely no right or wrong answers!  Also, if it is ok with you, we will audio-tape our discussion so that we can go back and listen to it at a later date for analysis. No one else will hear this tape and we will keep your personal information absolutely confidential and anonymous.  You may end the interview at any point, should you not want to continue.  Do you have any questions before I start the audio-recording?  **~ ~ ~**  Start tape. Say the date, ID code for the interviewee, recorder/interviewer name  *(e.g. Monday 23^rd^ September, Interview 1 with participant [ID code] and Nicola recording)*  Ok, let’s get started…  First, I am going to give you a brief definition of physical activity, so that we are clear on terms before we begin:  Physical activity is defined as any bodily movement, produced by skeletal muscles that requires energy expenditure. Physical activity encompasses all activities, at any intensity, performed during any time of the day or night. It includes exercise and incidental activity integrated into daily activity – this might include activities such as walking, running, cycling, swimming, or might even be activity within your day such as taking the stairs, walking to/from work etc…. . | |
| **PATIENT INFORMATION** | |
| Could you please confirm your consent to participate in the following telephone interview  Have you been given the opportunity to ask questions prior to your participation?  Have your questions been answered satisfactorily?  Could you also please confirm your age and date of birth?  Have you had a lung transplant? | |
| **CURRENT PHYSICAL ACTIVITY LEVELS** | |
| Let’s start with what you are doing currently, would you say that you are physically active?  Can you tell me about your past experiences with physical activity?  Are you aware of the current physical activity guidelines for PWCF? | **(prompt:** do you participate in PA often during a typical week? If yes, how often, what intensity, for how long?**)**  **(prompt:** positive/negative experiences?**)**  **(prompt:** how often, for how long, at what intensity, what type**)** |
| **MOTIVATORS** | |
| What motivates you to participate in physical activity?  What do you like or enjoy about physical activity? | (prompt: [a – social]routine, your health status, support from family and friends…  [b – personal] goal-setting, self-motivation, feeling better in yourself…  [c – access] access to gym facilities, healthcare professionals…) |
| **BARRIERS** | |
| In general, what prevents you from participating in physical activity? | **(prompt:** what challenges you, why don’t you like to participate in physical activity, what would stop you**)**  **(prompt:** time, lack of energy, lack of willpower, fear of injury/exacerbation, lack of ability, lack of access to facilities…**)**  **(prompt:** lack of support – HCP/family/friends, lack of HCP prescription/follow-up, transport, financial burden, perceptions of self – “I am too ill”**)** |
| **STRATEGIES TO IMPROVE ENJOYMENT / ADHERENCE** | |
| If you were to give other PWCF advice on how to be more physical active, what would you say?  Now, in your opinion, what would make participating in physical activity more appealing to you?  Do your healthcare providers discuss physical activity with you?  How supportive were your HCPs in relation to physical activity?  What could your HCP do to help you to increase your physical activity levels?  What would you like to see in a physical activity program, that would make you more inclined to incorporate it into your daily life and maintain it in the long term? | **(prompt:** if you were to tell them something that would empower them to be physically active and to more importantly **maintain** that behaviour…**)**  **(prompt:** what would make physical activity easier for you to participate in; what would make physical activity more enjoyable for you; what would make you adhere to a physical activity program more?**)**  **(prompt:** Regularly? In great detail? Prescribe?**)**  **(prompt:** spoke about it at every visit, spoke about it often, rarely spoke about it, only spoke about it when I brought it up**)**  **(prompt:** would your HCP monitor your physical activity?**)**  **(prompt:** physical activity broken up into smaller bouts throughout the day, rather than one long strenuous session/ technology/ hardcopy manual/ home-based/ community-based**)** |
